# High School Students' Online Learning Ineffectiveness in Experimental Courses During the COVID-19 Pandemic

**DOI:** 10.3389/fpsyg.2021.738695

**Published:** 2021-08-31

**Authors:** Jon-Chao Hong, Yue Liu, Yinsheng Liu, Li Zhao

**Affiliations:** ^1^Institute for Research Excellence in Learning Sciences, Department of Industrial Education, National Taiwan Normal University, Taipei, Taiwan; ^2^School of Education Science, Nanjing Normal University, Nanjing, China

**Keywords:** high school students, online learning ineffectiveness, online experimental courses, gender, COVID-19 pandemic

## Abstract

Due to the COVID-19 pandemic, online learning has been adopted in all stages of education. This sudden change from traditional learning to 100% online learning may affect students' learning effectiveness, especially in experimental courses. However, there has been little discussion of experimental courses conducted entirely through online learning. To address this gap, the present study investigated factors affecting high school students' online learning ineffectiveness (OLI) in online experimental courses, particularly online science experimental courses. The role of gender was also explored to understand whether it affects participants' OLI. An ANOVA was conducted to analyze the data from a survey of 347 online learners in high schools. The results indicated that the number of online experimental courses and the duration of online hands-on learning were negatively related to the high school students' OLI. Meanwhile, the study found that the high school participants' OLI differed by gender, with female students more likely than males to have OLI in the context of online learning. The results of this study can provide a reference for teachers who conduct online experimental courses and wish to improve their online teaching, not only during the COVID-19 lockdown, but also in other pandemic periods.

## Introduction

The Internet plays an important role in supporting remote work, online learning, online collaboration, and so on (Favale et al., 2020). Online learning and training make full use of the advantages brought by the innovation of Internet technology, breaking through the constraints of time and space, and constructing a new learning model which differs from face-to-face learning (Panigrahi et al., 2018). When students study via an online learning platform used as a virtual classroom, they can interact with teachers freely (Ganesh et al., 2015). However, scholars have pointed out that experimental courses which involve practical learning have often been implemented in face-to-face classrooms or laboratories, whereas the vast majority of online courses lack hands-on activities requiring experimental operations. It is, however, actually possible to complete online experimental courses with a set of components and a single-board microcontroller that are not especially costly (Burford and Gregory, 2002). Accordingly, during the COVID-19 pandemic, students in all high schools were required to engage in both theoretical and hands-on learning in online experimental courses, such as electronics courses, biochemistry laboratory courses, meaningful online extracurricular activities, information technology courses, and so on. Meanwhile, due to some special factors, online experimental courses have faced some difficulties during the COVID-19 pandemic. For example, due to the change in the delivery of the course materials, students might not be able to carry out hands-on activities as expected (Ghaemi and Potvin, 2021). Since the effects of those experimental courses delivered via online platforms during the COVID-19 pandemic have not yet been studied extensively, the present study explored the students' online learning ineffectiveness (OLI) in experimental courses in this context.

Many studies have focused on college students' online learning. For example, Kumari et al. (2020) proposed that the adoption of new technologies in online courses was able to boost graduate students' satisfaction with their competitive examinations, and encouraged participants to improve their inquiries in forums and with feedback on concepts and time management. In addition, Hong et al. (2016) found that using YouTube to learn guitar could facilitate college students' procedural knowledge learning. However, previous studies have also explored the impact of instructor, learner, and course factors on students' learning outcomes in K-12 online education (Zheng et al., 2020). Considering the COVID-19 pandemic, can online practice replace offline hands-on practice? The effectiveness of learning in experimental courses via the Internet was the focus of this study. Thus, the present study recruited high school students in order to explore the factors affecting their OLI related to experimental courses during the COVID-19 lockdown.

Previous studies have shown that there are gender differences in problems associated with media-related perceived attention and in the attention self-regulatory strategies adopted by learners in online learning (Wu and Cheng, 2018). However, Cuadrado-García et al. (2010) performed one-way and inter-individual factor ANOVA analyses and found that there was little difference in the motivation and satisfaction of male and female students. In addition to exploring the gender differences in engaging in online courses, the differences relating to students' online learning achievement should also be investigated (Martin et al., 2018). Thus, the present study took the number of online experimental courses students had to enroll in this semester and the duration of online hands-on learning for every school day as the factors to examine their correlation with the learners' OLI. Few studies have explored the gender influence of high school students' online study burden on their OLI in the context of their first-time 100% engagement in online learning. Therefore, the current study discusses OLI across genders mediated by the number of online experimental courses and the duration of online hands-on learning when high school students were engaging in online experimental courses. Accordingly, the purpose of the present study was to understand the impact of behavioral engagement on learning ineffectiveness, and whether the impact was gender-specific among high school students during the COVID-19 pandemic. The results of this study can provide an insight into how teachers can enhance students' learning engagement by changing the number and duration of online experimental courses and then promoting students' learning effectiveness during and after the coronavirus lockdown.

## Theoretical Background

An affordance, as defined by Gibson (1979), refers to the opportunity for action available to an individual based on characteristics of their environment (e.g., experiment lab). Successful action requires accurate movement corresponding to the environmental affordances. If an individual incorrectly perceives an action to be afforded, they may either attempt an action that is not possible or make a mistake in a science experiment. That is, improper action related to affordances may increase the risk associated with an experimental action, which could have substantial implications for an individual to safely and successfully execute the task requirements (Cordovil et al., 2015). Exposure to the factors influence successful action in a science experiment, for example, individual difference can compromise affordance engagement. Considering affordance theory, the science laboratory and equipment can afford students to take in science learning, the effectiveness of which is based on the individual learner's characteristics; thus, the present study took gender as a perspective of individual difference to explore how students perceived engaging themselves, and their learning ineffectiveness in online science experimental courses.

### Online Experimental Courses

Hands-on means that students gain experience by operating scientific instruments, hands-on processing, and observing scientific processes or objects (Rutherford, 1993). Therefore, we define experimental courses as those courses that apply the hands-on activity model which allows students to deepen their understanding and perceptions of knowledge through hands-on activities. Commonly found in high school courses such as biology, chemistry, physics, and science technology, this kind of course is not only fun, but also stimulates students' curiosity and creativity.

Many empirical studies have shown that carrying out experimental courses in subject teaching has a positive effect on promoting students' learning (Zhao et al., 2021a). For example, Thompson and Soyibo (2002) conducted a controlled experiment in chemistry classes. In the experimental group, the teaching of chemical electrolysis was carried out in the form of a mixture of teacher demonstrations and students' practice, while in the control group, the teacher demonstrations completely replaced students' hands-on practice. The results indicated that the experimental group showed more positive attitudes toward learning chemistry than the control group.

In normal teaching conditions, experimental courses are carried out in special environments, including laboratories, computer rooms, etc., but during the COVID-19 pandemic, many experimental courses have had to adopt the mode of online learning. This kind of online hands-on course combines theoretical interpretation and hands-on activities in the form of video courses and practice to help students acquire knowledge and become familiar with the skills. Thus, online experimental courses during the Covid-19 pandemic period are a focus of this study.

### Behavioral Engagement: The Number of Courses and the Duration of Online Learning

Visser (2001) defined learning engagement as the process of involving learners in “continuous dialogue with the human, social, biological and physical environment, so as to generate intelligent behavior to interact constructively with change” (p. 453). More specifically, Ally (2004) defined online learning engagement as “the use of the Internet to access learning materials; to interact with the content, instructor, and other learners; and to obtain support during the learning process, in order to acquire knowledge, to construct personal meaning, and to grow from the learning experience” (p. 7). Moreover, learning engagement, which emphasizes students' active participation in learning activities and completion of learning tasks, can be divided into three different types of engagement, specifically cognitive, behavioral, and emotional engagement. Hu et al. (2016) found that there is a need to understand how different environmental stimuli influence an individual's behavioral, cognitive, or effective engagement, in turn resulting in learning responses. Some studies have shown that among the three types of learning engagement, behavioral engagement is the more important factor to help learners achieve learning effectiveness (Fredricks et al., 2004; Lei et al., 2018). This school-related behavioral engagement can further improve student achievement. Accordingly, to explore the engagement in online experimental courses, the present study shifted the focus away from the study of how cognitive and emotional engagement intervene to enable high school students to engage with and to be seen to be (more or less) effective. Rather, it emphasized two relatively neglected features of these sorts of behavioral engagement: the number of online experimental courses and the duration of online hands-on learning.

According to Xiong et al. (2015), learning engagement in MOOCs can be measured by the number of lecture videos watched, forum posts made, quizzes taken, and tasks completed. MOOCs are a type of learning modality in online learning, so we can also measure students' behavioral engagement in online learning by measuring the number of online courses and the duration of online learning during the COVID-19 lockdown. What is more, the number of online experimental courses presents the nature of how often a high school student engages in online learning during a semester, while the duration of online hands-on learning enables us to understand how a high school student accumulates knowledge in the subjects. In line with this, these two types of engagement help us to elaborate on the concept of behavioral engagement in online experimental courses. Thus, the behavioral engagement of high school students during the Covid-19 pandemic period is another focus of this study.

### Online Learning Ineffectiveness

Online learning helps learners overcome the limitations of time and place. Behavioral engagement emphasizes the time and energy that learners devote to online learning courses in order to achieve the desired learning effectiveness (Hong et al., 2021). Most studies agree that it is debatable whether using online learning systems can achieve the same learning effectiveness as traditional learning (Pye et al., 2015).

Students' acquisition of learning outcomes will directly affect the success of the implementation of online learning (Panigrahi et al., 2018; Pinto et al., 2018). Researchers have found that emerging adolescents attempt to link with a self-perception bias. This is a “darker” feature of young people's psychology which links to their prejudice in attributing lower achievement outcomes to external factors (Anderson and Cheers, 2018). Moreover, adolescents have a tendency to “increase their endorsement of self-focused values and decrease their valuation of other-focused” behavior (Daniel and Benish-Weisman, 2019, p. 620). Due to their particular response bias, Hong et al. (2021) observed that young participants tend to self-report perceptions of ineffectiveness. Although self-reporting “ineffectiveness” is not common, from the perspective of the quality of the data, the present study considered that negative performance feedback could interfere with the achievement of stressful goals, which could in turn threaten the individual's ego and result in negative effect, thus creating negative value perceptions (Kluger and DeNisi, 1996). Kuhbandner et al. (2010) pointed out the role of negative effect in the negative “mirroring” of other actions. Considering this, the current study targeted high school students who were adolescents and who had the ego tendency to evaluate their learning effectiveness from a negative point of view. The present study therefore adopted learning ineffectiveness as a way for high school students to express their perceptions of online hands-on practice. Balancing these two types of views, in the present study we chose to use OLI rather than learning effectiveness to design the model of students' online learning performance. Thus, this study explored online learning ineffectiveness during the COVID-19 pandemic period.

### Gender Difference in Online Hands-On Learning

The factors which significantly affect students' barriers to online learning include gender and the number of online experimental courses they take in one semester (Muilenburg and Berge, 2005). Most studies on online learning have examined whether there are gender differences in psychological changes or learning outcomes (Pinto et al., 2018). Many studies have explored the factors that influence online learning from the perspective of learning resources and technologies. For example, some have found a correlation between students' personality traits and their online learning (Bidjerano and Dai, 2007; Komarraju et al., 2011; Ghyasi et al., 2013; Mirhashemi and Goodarzi, 2014). A number of studies have highlighted the association between students' learning outcomes and gender in their online learning process and retention (e.g., McSporran and Young, 2001; Martin et al., 2018; Rizvi et al., 2019; Zhao et al., 2021b). Many studies have taken gender differences into account when studying learners' learning effectiveness, indicating that gender difference is a major area of interest in learning effectiveness research.

Therefore, whether there is a difference in online learning results remains to be studied as few studies have explored the gender differences in high school participants' OLI in experimental courses. Thus, the present study used differential analysis to accurately predict students' gender difference in online OLI. The results of multi-group partial least-squares analysis indicated that differences exist in the utility of e-learning within gender and Generations X, Y, and Z. As these differences may be difficult to ascertain when only the gender level is examined, other researchers have concluded that the gender gap is narrowing. Considering this, this study also explored the impact of gender differences on learners' online learning ineffectiveness.

## Methods

### Example of Online Experimental Courses

Here are some online science experimental courses (OSEC) that high school students in China need to take during the COVID-19 pandemic. For example, students enrolled in the Physics Experiment courses can acquire practical knowledge of “the characteristics of plane mirror imaging” online. Under the “learning by doing” online teaching model, students can follow the instructor to investigate the imaging characteristics of plane mirrors on their own with hands-on experiments, and acquire theoretical and procedural knowledge. However, this requires students to do preparatory work in advance, such as preparing relevant experimental equipment as well as preparatory work on theoretical knowledge.

Another example is a unit of the online hands-on course, “Taking out and replacing the chemicals” learned by students taking chemistry courses. This online hands-on course integrates learning content, operating steps, and a Chemistry laboratory, forming an integrated teaching mode of theory and practice. It effectively simulates and reproduces the process of both taking out and replacing chemical reagents.

It is worth noting that, due to differences in regions, schools, and hardware facilities, there is some variation in the number and duration of online experimental courses that students need to take. Based on affordance theory, participants were informed that online experimental courses were designed for them to involve and evaluate the learning effectiveness of online experimental courses offered by schools during the COVID-19 lockdown; however, the content of experimental courses that the students studied on their own via the Internet is not included in our discussion.

### Research Questions

According to the learning engagement studies (Ally, 2004; Hu et al., 2016; Lei et al., 2018), this study took the number of online experimental courses, the duration of online hands-on learning, and demographic characteristics (e.g., gender) into account to explore high school participants' OLI in experimental courses. The present study aimed to answer two questions: (1) Is there a gender difference in participants' OLI in experimental courses? and (2) Is there a mediating effect of the two types of engagement in online experimental courses—the number of online experimental courses and the duration of online hands-on learning—between gender and OLI?

### Hypotheses

The number of online experimental courses and duration of online learning are the outward manifestations of the behavioral engagement of high school students that we focus on in online learning. E-learning effectiveness can be improved with the assistance of online course applicability assessment (Ren et al., 2017). Students usually face some difficulties at the beginning of online learning that prevent them from feeling satisfied with their participation in online courses (Rabin et al., 2020). For example, during online learning, the most prominent disadvantage is that immediate feedback on students' performance is seldom provided, which hinders learners' learning progress (Oyekan et al., 2017). This is a serious disadvantage related to the perception of learning ineffectiveness when implementing online learning courses.

In terms of the duration of online learning, one of its great advantages is that students can learn anytime and anywhere without the constraints of time, space, or location (Coleman and Furnborough, 2010). Meanwhile, a previous study on mental fatigue indicated that cognitive endurance in perceived performance tends to disappear as learning time increases (Giboin and Wolff, 2019). Therefore, it is extremely important for students to manage the duration of their online hands-on learning. Another study confirmed that there is a positive correlation between college students' duration of engaging in online learning and their learning achievement (Credé and Phillips, 2011). Accordingly, these two types of behavioral engagement help us eliminate high school students' online learning ineffectiveness in online experimental courses. To address this issue, the following hypothesis was proposed:

H1: The high school students' behavioral engagement is negatively related to OLI.

Previous studies have found that students' performance in online learning has a strong relationship with their characteristics, especially in terms of gender (Muilenburg and Berge, 2005; Rizvi et al., 2019). A growing number of studies have indicated that gender has no significant or consistent association with learning outcomes (Vermunt, 2005; Greene et al., 2015). However, some researchers consider gender as one of the most significant predictors of students' performance (Kizilcec and Halawa, 2015; Diep et al., 2016). Studies have shown that females have higher overall performance in online learning than male students (Rizvi et al., 2019). To address the issue that results concerning the impact of gender on learning outcomes in online learning are inconsistent and uncertain, the current study aimed to identify whether there was a gender difference in the influence of engaging in online learning on students' OLI. Thus, the following hypothesis was proposed:

H2: The influence of behavioral engagement on high school students' OLI differs by gender.

### Data Collection

This study adopted purposive sampling, with 100 samples from each of five high schools (i.e., a total of 500). Respondents were Grade 2 students with 1.5 years of hands-on experience of science experiments prior to the outbreak of the COVID-19 pandemic. All of the students had taken online science experimental courses during the pandemic. In this case, they could compare the real practice to virtual practice to ensure their perception of learning effectiveness or ineffectiveness. Moreover, they were invited to complete the online questionnaire. The study was approved by the University of Nanjing Normal University Ethics Committee. The researchers signed a standard confidentiality form stating that no sensitive information would be passed on, and that participants' anonymity would be ensured. In the introductory sessions, the importance of maintaining confidentiality of personal information was stressed to students.

However, 142 incomplete questionnaires were deleted due to the missing value of the analysis data or the short response time. Therefore, a total of 347 responses could be used for the following data analysis. The participants consisted of 263 females (75.8%) and 84 males (24.2%).

Additionally, students were asked how much time they had spent studying online each day in recent weeks: most (57.1%, *n* = 198) had spent about 2 to 4 h on online learning every day, 15.3% (*n* = 53) had spent about 1 to 2 h, 17.5% (*n* = 61) responded with 4 to 6 h every day, and 10.1% (*n* = 35) of the students had spent over 6 h on online learning.

### Instrumentation

In order to be able to determine the participants' learning ineffectiveness, they were asked to respond to items about their at-home online learning. Those respondents who had undergone a sudden transition to studying online at home were asked to respond to eight negative statements related to their learning during the COVID-19 lockdown. In the second part of the questionnaire, there were eight items using a 5-point Likert scale ranging from 1 (*strongly disagree*) to 5 (*strongly agree*) for assessing their OLI. A sample item is: “Since the online learning, my ability to learn independently has decreased.”

Cronbach's alpha was used to assess the internal consistency reliability. Hair et al. (2010a) reported that internal consistency coefficients (Cronbach's alpha) should range from 0.72 to 0.86 as the threshold for scales. In the present study, Cronbach's alpha exceeded 0.7 for all items, indicating that the scale had a high degree of internal consistency.

### Data Analysis

All the data were analyzed using IBM SPSS Statistics, version 19.0. To test our hypotheses, two types of data analysis were conducted. First, a Pearson correlation analysis was used to investigate the correlations among the number of online experimental courses, the duration of online hands-on learning, and OLI. Secondly, this research adopted a one-way ANCOVA to investigate whether participants' OLI differed according to their gender.

## Results

### Reliability and Validity Analysis

In this study, SPSS 25.0 and AMOS 24.0 were used to conduct an internal consistency test to ensure the reliability and validity of the instrument. If the standardized load of an item is <0.5, the item should be deleted (Hair et al., 2010b). The standardized factor loading of each item exceeded 0.50 (OLI: 0.594 - 0.879), and the average variance extracted (AVE) of OLI (0.5908) was >0.5, which indicates that the measurement tool had acceptable convergent validity (Fornell and Larcker, 1981). Moreover, the Cronbach's alpha of OLI (0.919) was >0.9, which indicated that the questionnaire had high internal consistency. For a more accurate measurement, the composite reliability (CR) is the reliability of all measurement items. The CR value of OLI (0.9193) was >0.7, showing that the items of the instrument had high reliability (Hair et al., 2010b).

### Behavioral Engagement Analysis

Regarding OSEC, the mean and standard deviation (SD) of the number of online experimental courses (NOEC), the duration of online hands-on learning (DOHL) and OLI for the whole sample and the gender groups are presented in Table 1.

**Table 1:** Behavioral engagement analysis.

	**N**	**%**	**NOEC**	**DOHL**	**OLI**
Total	347		4.43 ± 1.13	2.22 ± 0.83	22.87 ± 7.25
Male	84	24.21	4.80 ± 1.23	2.51 ± 0.94	21.04 ± 8.07
Female	263	75.80	4.32 ± 1.07	2.13 ± 0.77	23.45 ± 6.89

### Correlation Analysis

The results of a Pearson correlation analysis among NOEC, DOHL, and OLI are shown in Table 2. OLI was negatively correlated with DOHL (*p* < 0.01) and NOEC (*p* < 0.05).

**Table 2:** Correlation analysis between OLI, DOHL, and NOHC.

**Variables**	**DOHL**	**NOEC**	**OLI**
DOHL	1	0.327^**^	−0.175^**^
		0.000	0.001
NOEC		1	−0.135^*^
			0.012
OLI			1

* *p < 0.05*,

** *p < 0.01*.

### Gender Difference in Learning Ineffectiveness

A one-way ANCOVA was performed considering gender as the independent variable and online learning ineffectiveness as the dependent variable. The results showed that there was a significant online learning ineffectiveness difference between males and females (*p* < 0.000); specifically, by comparing the mean values of males and females, it was found that females' learning ineffectiveness was higher than that of males in online learning.

Another one-way ANCOVA was performed to assess whether online learning effectiveness was affected by the curriculum-level factors (NOEC and DOHL) differing by the demographic characteristic (gender). Gender was included as the covariate in the analysis.

Firstly, the one-way ANCOVA conducted to analyze OLI revealed a significant difference in the duration of online hands-on learning after adjusting for gender [*F*_(3)_ = 3.629, *p* < 0.05, effect size η2 = 0.031]. However, the ANCOVA results for covariate gender were not significant (*p* > 0.05). Bonferroni-corrected, multiple *post hoc* comparison tests indicated that students who studied online for 1–2 h per day had higher OLI than students who studied online for 2–4 h per day (SE = 1.099, 95% CI = −2.527–1.798), students who studied online for 4–6 h per day (SE = 1.339, 95% CI = −1.279–3.989), and students who studied online for over 6 h per day (SE = 1.574, 95% CI = 0.754–6.947). Students who studied online for 2–4 h per day had higher OLI than both students who studied online for 4–6 h per day (SE = 1.044, 95% CI = −0.334–3.773) and students who studied online for over 6 h per day (SE = 1.328, 95% CI = 1.603–6.929). Students who studied online for 4–6 h per day had higher OLI than students who studied online for over 6 h per day (SE = 1.517, 95% CI = −0.4886–5.479).

Secondly, ANCOVA was conducted to investigate whether OLI changed with NOEC. Gender was included as a covariate in the analysis. The ANCOVA revealed that OLI did not differ by NOEC [*F*_(3)_ =1.280, *p* > 0.05, effect size η2 = 0.026]. There was a significant effect for the covariate gender [*F*_(1)_ = 5.264, *p* < 0.05].

### Verification of Hypotheses

The correlation analysis results found a correlation between OLI and the curriculum-level factors (NOEC and DOHL). The ANCOVA results revealed that OLI differed by gender. According to the results, all the hypotheses were supported.

According to the results of Pearson correlation analysis, H1 is supported: Behavioral engagement is negatively related to online OLI.

According to the ANCOVA results, participants' OLI in online learning partly differed by gender. The influence of NOEC on participants' OLI differed by gender. The mean OLI of the female participants in this study was 23.5 (SD: 6.89), which was higher than that of the males (X = 21.04; SD: 8.07). However, the influence of the duration of online learning on students' OLI did not change with gender; thus, H2 was partly supported.

## Discussion

The present study investigated the influence of the factors including NOEC and DOHL on participants' OLI in online learning. In addition, the study also explored whether the influences changed according to gender.

As different learning designs can influence individuals' cognitive or effective engagement, resulting in specific behavioral responses (Hu et al., 2016), this study aimed to understand how the extent of high school students' behavioral engagement in OSEC intervenes in the effectiveness/ineffectiveness of their engagement. The present study emphasized two relatively neglected features of these sorts of behavioral interventions: NOEC and DOHL. They were both negatively related to OLI. H1 was thus supported. In terms of the relationship between NOEC and participants' OLI, the results indicated that there was a negative significance. This finding was the same as that of the study of Rabin et al. (2020), in which learners who intended to complete only some parts of the course activities or who did not know how many parts of the course they intended to complete faced stronger barriers to satisfaction than those who intended to complete all of the course activities. That is, the more participants engaged in online learning, the less unsatisfied (i.e., the more satisfied) they were with their learning effectiveness. In terms of the relationship from the duration of online hands-on learning and participants' OLI, those high school students who spent more time on online hands-on learning would be less likely to be dissatisfied with online learning. This result is in line with a recent meta-analytical review which confirmed that college students' time engagement in online courses is positively related to their learning achievement (Credé and Phillips, 2011). Meanwhile, it was also supported by a previous study that indicated a negative significance in the effects of learning engagement on the relationships between perceived usefulness and learning performance (Jung and Lee, 2018; Galikyan and Admiraal, 2019). Therefore, lengthening students' duration of online hands-on learning enables them to be more whole-heartedly involved in the online hands-on learning, so that they can have a good sense of participation in the online learning and have reduced OLI.

For high school students, it is imperative to go through online theory courses to gain declarative knowledge. Similarly, online experimental courses are also imperative for some science experiments or practical activities during the COVID-19 lockdown. According to previous research, we divided learning engagement into three categories: emotional, cognitive, and behavioral (Fredricks et al., 2004; Sun and Rueda, 2012). Behavioral engagement, an important component of learning engagement, can be a direct reflection of students' satisfaction and acceptance of course rules and requirements. Our analysis of the relationship between students' behavioral engagement in online experimental courses and their learning ineffectiveness provides strong evidence for our schools and the relevant education authorities to improve online education at the course level.

With respect to gender differences, no obvious difference was found in OLI according to DOHL, but OLI affected by NOEC was found to differ by gender, indicating that H2 was partly supported. These results show coherence with the results of some previous studies (Cuadrado-García et al., 2010; Cai et al., 2017; Park et al., 2019). On the one hand, one previous study confirmed that gender difference plays a role as a moderator in the adoption of multimedia for learning (Park et al., 2019). Meanwhile, Cai et al. (2017) indicated that women also hold more favorable attitudes toward technology use than men. Based on the perspective of affordance theory, the reason for the gender difference in online learning ineffectiveness may be that there are differences in elicitation (Díaz et al., 2021), and adaptability (Drupp et al., 2020) to the learning environment which leads to a weaker sense of online learning ineffectiveness for males than for females. On the other hand, some other studies have not found any difference. Cuadrado-García et al. (2010) indicated that there are few differences between male and female students in their use of online learning and their motivation and satisfaction. However, the result of this study indicated that female students perceived higher OLI than male students. The reason behind this may lie in the fact that online learning organized in emergency situations makes students unable to adapt to it quickly, and at the same time, due to the limitation of online platforms and online learning resources, learners all felt a certain degree of online learning ineffectiveness.

## Conclusions

During the COVID-19 pandemic, high school courses including experimental courses have been taught online. In order to understand the factors that influence high school students' online OLI in experimental courses, this study explored the effect of NOEC and DOHL on OLI in experimental courses. On this basis, the gender difference of these effects has been discussed. The results showed that NOEC was negatively related to participants' OLI, indicating that the more courses there are, the more students will be able to interact with their teachers and peers. However, the results also show that there was a gender difference in the occurrence of OLI, with female students more likely to have OLI than males.

### Implications

This study firstly examined the impact of the online study burden, including NOEC students had to enroll in during one semester, and DOHL for every school day, on participants' OLI. The theoretical significance of this study is that it clarifies that NOEC and DOHL will affect the OLI of high school students in online learning, and validated that OLI did indeed differ according to students' gender.

The results show that DOHL is negatively related to high school participants' OLI, and there is no gender difference in the OLI of students participating in OSEC. To increase DOHL, students' knowledge and ability are continuously accumulated, which lays the foundation for their subsequent behavioral engagement. Both the increasing OSEC and DOHL can increase the participation of students in OSEC. Once they can have a good sense of participation, the OLI of high school students will be alleviated to some extent in their online learning.

The practical implication of the current study is that it provides some data support for teachers to understand students' engagement in OSEC from the perspective of the offline-to-online transition. That is, the findings can, to some extent, help teachers to design better offline experiment courses and to promote students' learning interest or to conduct some online and offline mixed experimental courses (i.e., by providing real materials without danger to students to experience science experiments). Thus, teachers can observe offline and give students timely guidance to help them overcome problems in experiments, and study the gender difference.

### Limitations and Future Study

Some limitations are addressed here. First, from the point of view of the source and number of samples, the participants included in this study all came from the same province, and it is difficult to guarantee that these samples covered all levels of high school education institutions. Therefore, the samples collected this time may not be representative of all high school students. In the future, additional and larger representative samples need to be collected to ensure that the conclusions of this study can apply to a wider range of population groups and countries.

Another weakness of this study is that there is no clear definition of OLI. In the questionnaire designed for this study, there are eight questions regarding OLI which may not reflect the students' possible OLI in online experiments in detail. This problem needs to be addressed in the future research. It is necessary to conduct a pre-test to ensure a more accurate understanding of any gap between real performance and the possible OLI of students in online experimental performance.

The third limitation of this study is the statistical analysis. ANCOVA was conducted on the sample size of 347 students, which is a quantitative method that tells us only whether differences are statistically significant or not. Therefore, conducting qualitative analysis will be a valuable contribution of future research.

## Data Availability Statement

The original contributions presented in the study are included in the article/supplementary material, further inquiries can be directed to the corresponding author.

## Author Contributions

J-CH conceived the original idea and reviewed and edited the manuscript. YuL and LZ wrote the manuscript. YiL contributed to the interpretation of the results. LZ supervised the project. All authors have read and agreed to the published version of the manuscript.

## Conflict of Interest

The authors declare that the research was conducted in the absence of any commercial or financial relationships that could be construed as a potential conflict of interest.

## Publisher's Note

All claims expressed in this article are solely those of the authors and do not necessarily represent those of their affiliated organizations, or those of the publisher, the editors and the reviewers. Any product that may be evaluated in this article, or claim that may be made by its manufacturer, is not guaranteed or endorsed by the publisher.
